# Identification, chromosomal arrangements and expression analyses of the evolutionarily conserved *prmt1* gene in chicken in comparison with its vertebrate paralogue *prmt8*

**DOI:** 10.1371/journal.pone.0185042

**Published:** 2017-09-21

**Authors:** Yi-Chun Wang, Chien-Wen Wang, Wen-Chang Lin, Yun-Jung Tsai, Chien-Ping Chang, Yu-Jen Lee, Min-Jon Lin, Chuan Li

**Affiliations:** 1 Department of Biomedical Sciences, Chung Shan Medical University, Taichung, Taiwan; 2 Department of Medical Research, Chung Shan Medical University Hospital, Taichung, Taiwan, ROC; 3 Institute of Biomedical Sciences, Academia Sinica, Taipei, Taiwan, ROC; Chang Gung University, TAIWAN

## Abstract

Nine protein arginine methyltransferases (PRMTs) are conserved in mammals and fish. Among these, PRMT1 is the major type I PRMT for asymmetric dimethylarginine (ADMA) formation and is the most conserved and widely distributed one. Two chicken *prmt1* splicing variants were assembled and confirmed by RT-PCR experiments. However, only two scaffolds containing single separate *prmt1* exon with high GC contents are present in the current chicken genome assembly. Besides, *prmt1* exons are scattered in separate small scaffolds in most avian species. Complete *prmt1* gene has only been predicted from two falcon species with few neighboring genes. Crocodilians are considered close to the common ancestor shared by crocodilians and birds. The gene arrangements around *prmt1* in American alligator are different from that in birds but are largely conserved in human. Orthologues of genes in a large segment of human chromosomal 19 around *PRMT1* are missing or not assigned to the current chicken chromosomes. In comparison, *prmt8*, the *prmt1* paralogue, is on chicken chromosome 1 with the gene arrangements downstream of *prmt8* highly conserved in birds, crocodilians, and human. However, the ones upstream vary greatly in birds. Biochemically, we found that though *prmt1* transcripts were detected, limited or none PRMT1 protein was present in chicken tissues. Moreover, a much higher level of PRMT8 protein was detected in chicken brain than in mouse brain. While PRMT8 is brain specific in other vertebrate species studied, low level of PRMT8 was present in chicken but not mouse liver and muscle. We also showed that the ADMA level in chicken was similar to that in mouse. This study provides the critical information of chicken PRMT1 and PRMT8 for future analyses of the function of protein arginine methyltransferases in birds.

## Introduction

Protein arginine methylation is a posttranslational modification playing multiple roles in signal transduction, epigenetic regulation, DNA repair, RNA processing and developmental regulations. Protein arginine methyltransferases (PRMTs) catalyzing the reaction are divided according to the way they transfer methyl groups to specific guanidino nitrogen atoms of arginines. In mammals, PRMT1, 2, 3, 4, 6 and 8 catalyzing the formation of asymmetric ω-*N*^*G*^, *N*^*G*^ dimethylarginine (ADMA) belong to type I [1, 2]. Type II PRMT5 and PRMT9 catalyze the formation of symmetric ω-*N*^*G*^, *N*^*G’*^ dimethylarginine (SDMA) [3]. PRMT7, the only type III PRMT, catalyzes the formation of ω- *N*^*G*^ monomethylarginine (MMA) [1, 2].

Orthologues of all nine mammalian PRMTs can be identified in zebrafish. Interestingly, of the nine vertebrate PRMTs, PRMT2 and PRMT6 are lost in reptiles. Outside vertebrates, amphioxus (*Branchiostoma floridae*, a cephalochordate) has homologues of all mammalian PRMTs except PRMT8 [4]. PRMT8 has conserved amino acid sequences as well as gene structures with PRMT1 from fish to human, thus is a vertebrate–restricted paralogue of PRMT1 [5].

PRMT1 is the most evolutionary conserved and ubiquitously expressed PRMT. PRMT1 accounts for the majority of type I PRMT activity [6]. It catalyzes the methylation of a plethora of proteins including many nucleic acid binding proteins such as fibrillarin [7], hnRNPA1 [8], SERBP1 [9] and CNBP [10] with glycine and arginine rich motifs or the RGG sequences. PRMT1 can methylate histone H4 at the third arginine residue (H4R3) and thus can act as a coactivator for histone code and epigenetic regulation [11, 12]. Mice with *prmt1* null mutation died after implantation [13]. Zebrafish embryos with *prmt1* knockdown by antisense morpholino (AMO) injection showed delayed growth and defects during gastrulation [14]. These results indicate the critical roles of PRMT1 in the development of mammals and fish.

The major difference between PRMT1 and PRMT8 is that PRMT8 contains an extra N-terminus for about 60–90 amino acids. PRMT8 is the only PRMT with tissue (neuronal) specific expression [15]. Mouse *prmt8* mutants grew and reproduced normally but displayed abnormal motor behaviors [16]. We conducted zebrafish *prmt8* knockdown and rescue experiments and showed that *prmt8*, depending on its specific N-terminus, plays important roles in embryogenesis and brain development non-overlapping with *prmt1* [17].

Avian genomes with large segmental deletions and gene loss are more compact than other vertebrate genomes [18]. The constrained avian genome with fewer repetitive elements and less non-coding DNA has been proposed to be a key adaptation to reduce the metabolic costs of powered flight [19]. The common ancestor of birds experienced large segmental deletions most probably through the chromosomal fragmentation events that lead to the microchromosomal formation in modern birds [18]. Furthermore, less protein-coding genes (~15,000 for chicken) are predicted in avian compared to that in mammals (~20000 for human). Comparative genome analyses demonstrated fewer gene family members in birds than in other vertebrates [20]. It is known that most of the genes missing in birds are clustered in blocks with the same chromosomal arrangements in lizard and human [21]. Two most extensively studied type I PRMT family members, PRMT1 and PRMT4 (CARM1), are on human chromosome19 that contains large chromosomal segments missing in birds. In the recently released Gallus_gallus-5.0 genome assembly, *carm1* was classified as mammalian or lizard genes that are found in sequenced birds but not chicken [22]. Though *prmt1* is not on the missing gene lists in these studies, its chromosomal localization is not clear. Furthermore, its highly conserved paralogue *prmt8* might complicate the annotation. We thus are interested in the existence of the *prmt1* gene in birds.

Birds and crocodilians are the only extant members of archosaurs (‘ruling reptile’), a monophyletic group containing the extinct dinosaurs [18]. As crocodilian genomes evolve extremely slow [23], crocodilians should be close to the common ancestor shared by crocodilians and birds. To trace *prmt1*, we compared the chromosomal neighbors of *prmt1* and *prmt8* in human, avian species including chicken, falcons and ground tit, as well as reptiles including lizard and crocodilians. We further analyzed the syntenic gene arrangement of *prmt1* and *prmt8* in typical vertebrate branches for the evolution of gene arrangements in vertebrates.

In addition, in this study, we also investigated the level of RNA and protein products of both *prmt1* and *prmt8*, the level of ADMA-containing proteins, as well as the distribution of the PRMTs in subcellular fractions of chicken brain extracts. Basic biochemical analyses can provide more information to evaluate the chicken PRMTs.

## Materials and methods

### Establish the syntenic arrangement of the genes encoding PRMT1 and PRMT8 in representative vertebrate species

The chicken (*Gallus gallus*) *prmt1* coding sequence was assembled manually from available transcriptome databases. The sequence was used to BLAST avian databases for orthologues. Syntenic gene arrangement around *prmt1* and *prmt8* in human (*Homo sapiens*), American alligator (*Alligator mississippiensis*), green anole (*Anolis carolinensis*), and avian species including chicken (*Gallus gallus*), saker falcon (*Falco cherrug*), peregrine falcon (*Falco peregrinus*) and Tibetan ground tit (*Pseudopodoces humilis*) were retrieved from NCBI.

### Animal tissue extract preparation

Mouse tissues were from mice sacrificed for a study of female hormones on neuromuscular transmission with the permission from the Institutional Animal Care and Use Committee, Chung Shan Medical University. Chicken tissues were freshly prepared from chicken slaughtered in a local market. Fresh tissues were stored at −80°C. All tissues were weighed, washed two times with phosphate buffered saline (PBS), cut into small slices, and then resuspended into 5× (vol/wt) TRIzol reagent (Molecular Research Center, Inc.) for RNA preparation or in 3× (vol/wt) lysis buffer [50 mM Tris-HCl, pH7.4, 150 mM NaCl, 1 mM EDTA, 1% Triton X-100, 10 mM NaF, 1x complete protease inhibitor (Roche)] for protein extract preparation. The protein concentration in each fraction was determined using the BCA reagent (Pierce) with bovine serum albumin as the standard protein.

### mRNA expression analyses by RT-PCR

Total RNA was isolated from chicken and mouse tissues, including brain, liver and muscle by TRIzol reagent (Molecular Research Center, Inc.). First strand cDNA was synthesized from 3 μg of total RNA by SuperScript III Reverse Transcriptase (Invitrogen). RT-PCR was performed with the primer set: mouse *prmt1* forward (5' GGAGCTGCCCGTGGAGAA 3') and mouse *prmt1* reverse (5' GAGAAGCCGGTCCTCTTGTG 3’), mouse *prmt8* forward (5'

AGCAAGTGGTGACCAATGCCTG 3') and mouse *prmt8* reverse (5' GGACATAGTCGTTGCGCTGGAT 3'), chicken *prmt1*-1 forward (5' GGAGATGCTGAAGGATGAGG 3') and chicken *prmt1*-1 reverse (5' GACGATCTTGACGGCGTAAT 3'), chicken *prmt1*-2 forward (5' GGCGAACTGCATCATGGA 3') and chicken *prmt1*-2 reverse (5' TTGGCAGCAAACATGCATAG 3'), chicken *prmt8* forward (5' TCCCAGACTCCTCAGCCTAC 3') and chicken *prmt8* reverse (5' AGGATTCCTGTGCCACTTCC 3'). The cDNA of glyceraldehyde 3-phosphate dehydrogenase (*gapdh*) was amplified with the primer set: mouse *gapdh* forward (5' AACTTTGGCATTGTGGAAGG 3') and mouse *gapdh* reverse (5' ACACATTGGGGGTAGGAACA 3'), and chicken *gapdh* forward (5' GTGAAAGTCGGAGTCAACGG 3') and chicken *gapdh* reverse (5' ACAGTGCCCTTGAAGTGTCC 3') as controls. Amplified cDNA products were resolved by agarose electrophoresis and visualized by ethidium bromide staining.

### Isolation of mouse and chicken tissue histones

Nuclei were collected by centrifugation at 17, 530 xg at 4°C for 20 min. Histones were acid extracted by shaking in 0.2 M sulfuric acid for 1 h at 4°C. After centrifugation, histones were precipitated with ethanol at −20°C overnight, washed once with ethanol, and resuspended in distilled water.

### Fractionation of mouse and chicken brain extract

Brain washed with PBS was cut into small slices, re-suspended in 3× (vol/wt) homogenization buffer (0.32 M sucrose, 20 mM Tris, pH 7.3, 5 mM MgCl_2_, 1 mM PMSF, 1 mg/ml aprotinin, 1 μg/ml pepstatin, 1 μg/ml leupeptin) and homogenized with a Teflon pestle with about 10 strokes. The fractionation scheme was illustrated in S1 Fig following the procedures of Hung et al [24]. Briefly, the homogenate (S0) was centrifuged at 800 ×g for 10 min and the pellet was P1. The supernatant S1 was further centrifuged at 9,200 ×g for 15 min. The supernatant and pellet fractions were designated as S2 and P2. Ultracentrifugation of the S2 fraction at 130,000 ×g for 1 h resulted in the supernatant S3 (cytosolic) and pellet P3 (membrane) fraction. Part of the P3 was re-suspended in lysis buffer with 0.5 M KCl and subjected to ultracentrifuge again. The supernatant and the pellet fractions were S4 (high-salt-stripped soluble) and P4 (high-salt-stripped membrane pellet), respectively.

### Western blot analyses

Samples were separated by SDS–PAGE and transferred to nitrocellulose membranes (Gelman Science). The membranes were blocked with 5–7% skimmed milk powder in TTBS (10 mM Tris–HCl, pH7.5; 100 mM NaCl; 0.1% tween 20) for 30 min, incubated with primary antibodies (1:1000 for ADMA, 1:1000 for SDMA, 1:1000 for MMA from Cell Signaling; 1:1000 for anti-histone H4, 1:1000 for anti-histone H4 R3Me2, and1:2000 for anti-PRMT1 (07–404) from Millipore/Upstate or 1:1000 for anti-PRMT1 from Abcam (MAT-B12, ab7027), 1:2000 for anti-PRMT8 from Millipore or from Abiocode; 1:10000 for anti-PRMT3 and 1:2000 for anti-FMRP from abcam; 1:500 for anti-hnRNP A2/B1 and 1:2000 for anti-GAPDH from Santa Cruz) overnight, washed three times in TTBS, then incubated with secondary antibody for 1 h. Chemiluminescent detection was performed using the Supersignal kit (Pierce) or VisGlow Chemiluminescent Substrate, HRP (Visual Protein, Taiwan).

## Results

### Assembly of chicken *prmt1* transcripts from the database

**Fig 1:**
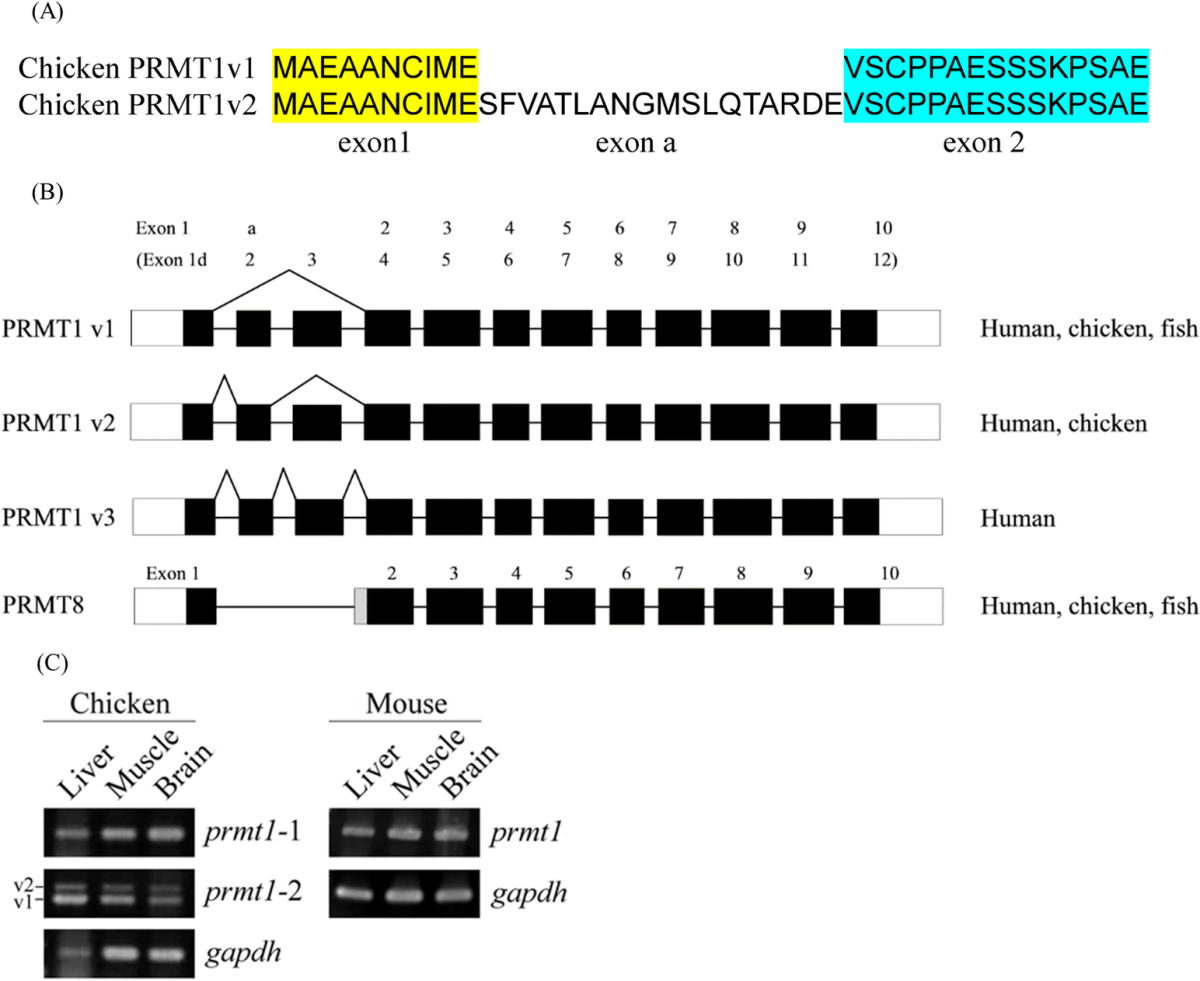
N-terminal amino acid sequences, genomic configuration and expression of the *prmt1* gene in chicken. (A) The N-terminal amino acid sequences of chicken PRMT1 v1 and v2. (B) Schematic illustration of the three major *prmt1* variants due to alternative splicing at the 5’ end in human, chicken and zebrafish. The gene structure of *prmt8* very close to *prmt1* v1 is also shown. The nomenclature of the exons according to human *PRMT1* by Goulet et al. [26] is shown in the parenthesis. (C) Expression of *prmt1* variants in chicken analyzed by RT-PCR. The products amplified by the primers for the constitutive exons (*prmt1*-1) and the products amplified by the primer set encompassing the alternative exon (*prmt1*-2) are shown. Mouse *prmt1* transcripts were also analyzed in comparison. *Gapdh* indicates the RT-PCR product of glyceraldehyde 3-phosphate dehydrogenase as the control. NC: negative control. L: liver, M: muscle, B: brain.

To investigate PRMT1 in birds, we first conducted tBLASTn with human PRMT1 sequence against the chicken genome and no targets could be identified. Nevertheless, *prmt1* genes are present in peregrine falcon (*Falco peregrinus*) and saker falcon (*Falco cherrug*), whose genomes are assembled with more than 100-fold coverage and have about 16,200 genes predicted [25]. The predicted proteins share high sequence identity with human PRMT1. The avian genome is of a high evolutionary stasis at the level of nucleotide sequences, gene synteny and chromosome structures [18]. It is thus likely that the *prmt1* orthologue is present in chicken but missed in the current chicken genome assembly. We then used the human or falcon *prmt1* as the query to conduct nucleotide BLAST against the chicken or avian NCBI nr database. Even though there were few target hits, among them were two chicken cDNA entries (CR524297 and BX930603). We further assembled full-length chicken cDNA sequences with available EST and TSA sequences to fill the ends. The predicted sequences are consistent with two splicing variants with or without an alternative exon at the 5’ region between the 1^st^ and 2^nd^ constitutive exons (Fig 1A). Six ESTs support the variant without the alternative exon and two ESTs support the other variant with the alternative exon. Due to alternative splicing of the 5’ exons, there are at least seven different human *PRMT1* transcripts reported, with v1 and v2 the most abundant ones [26]. The assembled chicken variant 1 and variant 2 with the accession number BK010274 and BK010275 are similar to the human v1 and v2 variants, with the first chicken exon corresponding to human e1d, the alternative exon corresponding to human e2 and the 2^nd^ constitutive exon corresponding to exon 4. In comparison, in zebrafish only one *prmt1* transcript had been identified [14]. The illustration of the gene structure that is also conserved in the paralogous *prmt8* gene is shown in Fig 1B.

We then validated the expression of chicken *prmt1* by RT-PCR analyses. We prepared RNA from chicken tissues and designed primers. The first primer set to amplify the constitutive exons (*prmt1*-1) could detect signals in brain, liver, and muscle. Besides, with the primer set encompassing the alternative exon (*prmt1*-2), two RT-PCR products corresponding to the two splicing variants could be detected (Fig 1C). The signals of the variant without the alternative exon appeared to be stronger, consistent with more supporting ESTs. We also showed the expression of mouse *prmt1* transcripts in three tissues.

We then designed primers according to the assembled sequences to amplify the whole coding region of *prmt1* and confirmed the coding sequences of v1 (accession number MF630622). A T/C single nucleotide polymorphism is present at nucleotide from the ATG start codon. We also used the chicken *prmt1* cDNA sequence to conduct nucleotide BLAST of the chicken genome. In chicken, two hits corresponding to exon 1 (chrUn_Scaffold23446) and exon 8 (chrUn_Scaffold23978) were identified from the newly released Gallus_gallus-5.0. They are separate small scaffolds (2017 bp and 1863 bp) of unknown chromosomes with high GC content (0.7297 and 0.6334) and long GC stretches.

### Most avian *prmt1* genes are poorly assembled but assembly of other avian *prmt1* revealed high sequence conservation

**Fig 2:**
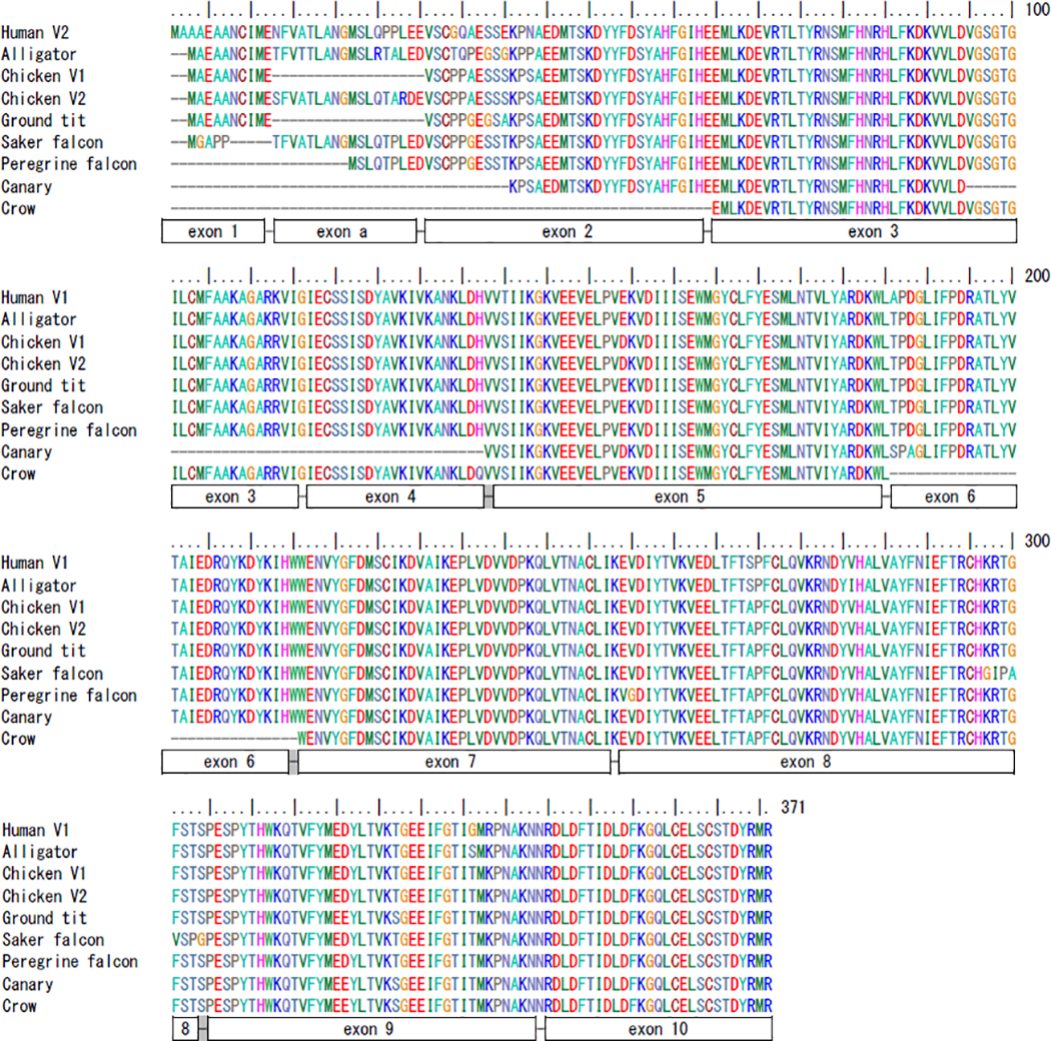
Alignments of assembled avian PRMT1 protein sequences. The predicted protein sequences from assembled chicken, ground tit, saker falcon, peregrine falcon, canary and crow *prmt1* coding sequences are aligned with human and alligator PRMT1 by ClustalW. We added a nucleotide G that is conserved in all other species to fill the one nucleotide gap at the end of exon 5 of ground tit *prmt1* for sequence translation. The amino acids encoded by different exons are illustrated by the underneath boxes. The position of the short line connecting the exons indicating the end of the previous exon or at the amino acids encoded by both exons (shaded).

We used the assembled chicken *prmt1* cDNA as the query to BLAST avian genomes to detect more avian *prmt1* genes. Forty-three hits from twenty-two avian species could be identified with the default parameters. The identities of the nucleotide sequences are mostly higher than 90%. The only target with the sequence identity lower than 85% (83%, corresponding partial exon sequence) is from *prmt8*. Of the rest forty-two targets, thirty correspond to a single exon of *prmt1* and twenty-one are in scaffolds shorter than 1000 bp, indicating that they are from poorly assembled chromosomal fragments. Among the hits, 17 contain exon 8 and none contain exon 4, the smallest constitutive exon (S1 Table). A scaffold that contains six *prmt1* exons (2, 5, 7, 8, 9, 10) was detected in common canary *Serinus canaria*. Though exon 3, 4 and 6 may be missed in the scaffold, partial sequences of exon 3 can be identified in another scaffold. We then manually assembled the canary *prmt1* based on these sequence. We also assembled and re-annotated the *prmt1* from four scaffolds (with exon 3, exon 5 and 7, exon 8 and 9, exon10) from American crow *Corvus brachyrhynchos* and three scaffolds from Tibetan ground tit *Pseudopodoces humilis* (exon3, exon 5, and exon 7, 8, 9) together with available predicted mRNA sequences (S1 File). Sequence alignments of the predicted protein-coding region of the assembled avian *prmt1* sequences showed high conservation (S2 File). We also aligned the four avian PRMT1 sequences assembled in this study with predicted falcon PRMT1 sequences as shown in Fig 2. Though some of the sequences are incomplete, it is clear that they are highly conserved with few residue variations. The N-terminal sequence of PRMT1 encoded by exon 1 varies in different species and may be misannotated by automatic prediction. Almost perfect alignments of the N-termini from the predicted chicken and ground tit PRMT1 with that from human and alligator PRMT1 suggest that the sequences are highly reliable.

### Comparison of the gene arrangements close to *prmt1* in avian species and in sauropsids

**Fig 3:**
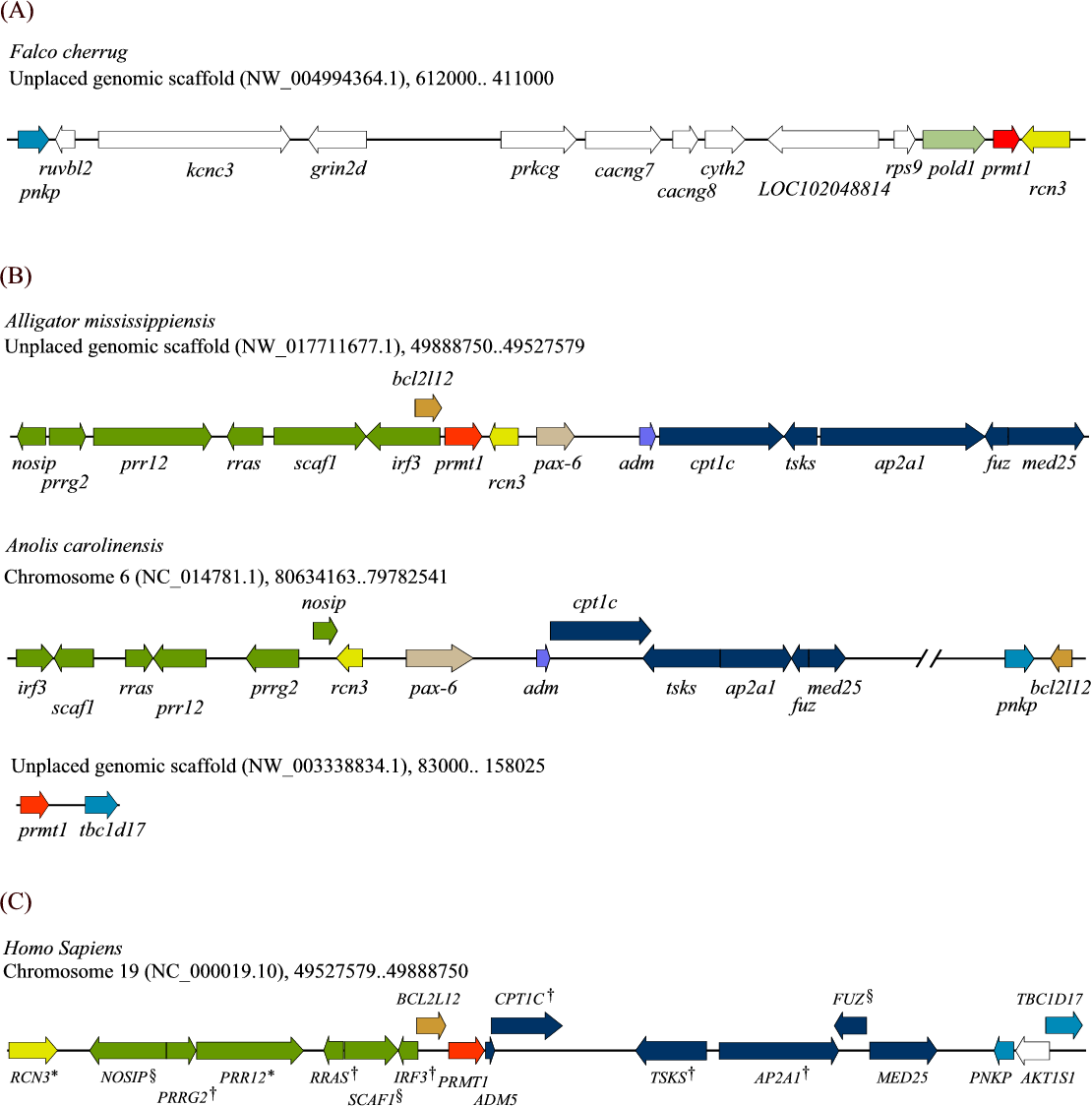
Syntenic gene arrangements near *prmt1* in amniotes. **(**A) Schematic diagram of the available gene arrangements around *prmt1* in saker falcon. (B) Schematic diagram of the available gene arrangement around *prmt1* in American alligator and green anole. (C) Schematic diagram of the available gene arrangement around *PRMT1* in human. Genes that have been classified by the Gallus_gallus-5.0 genome assembly as missing in chicken are marked with*, missing in avian are marked with †, genes assigned to unknown chromosome are marked with §. The length and the direction of the arrows were diagramed according to the size and transcription directions of the genes. Gaps indicate the presence of other genes between the genes shown in the diagram. Genes in the upstream or downstream syntenic blocks conserved in alligator, green anole and human are shown in dark green and blue respectively. Besides genes in these blocks, orthologous genes in different species are shown in the same color.

As indicated in the previous sections, most of the avian *prmt1* genes are now fragmented in small scaffolds from unknown chromosomes. We could obtain syntenic gene arrangements around *prmt1* only in saker falcon with 21 genes in the scaffold containing *prmt1* (Fig 3A). We then tried to trace the gene arrangement neighboring *prmt1* through evolution. We used American alligator (*Alligator mississippiensis*; a reptile as a non-avian sauropsid), a crocodilian with the best assembled genome, to trace the syntenic block arrangement in the common ancestors of archosaurs. Fifteen genes are in the same assembly with *prmt1* in alligator as shown in Fig 3B. Among them, only *rcn3* is a direct *prmt1* downstream neighbor with the same opposite direction in falcons.

Human *PRMT1* is on chromosome 19, the chromosome that has the highest gene density and contains the majority of the missing blocks in the assembled avian genomes. The *prmt1* gene in alligator clusters with seven orthologous genes directly upstream of human *PRMT1* in the *NOSIP-PRRG2-PRR12*-*RRAS-SCAF1-IRF3-BCL2L12-PRMT1* order (Fig 3C). The *rcn3* gene that is directly downstream of *prmt1* in alligator rearranged upstream of the cluster in human. Human orthologues of the five *prmt1* downstream genes *cpt1c-tsks-ap2a-fuz-med25* in alligator also clustered the same way on chromosome 19.

Missing chicken genes were reported to be clustered in syntenic blocks in lizard and human. To follow the possible chromosome rearrangements near *prmt1*, we also analyzed the genes in green anole (*Anolis carolinensis*), another model reptile. Most of the orthologues of human *PRMT1* neighbors are present on chromosome 6 of green anole. The upstream genes *nosip-prrg2-prr12-rras-scaf1-irf3* are linked and five downstream genes *cpt1c-tsks-ap2A-fuz-med25* are clustered together as in human (Fig 3B). The *rcn3-pax6-adm* arrangement is present in both alligator and green anole but not human or birds and is connected directly with the *cpt1c* to *med25* block. However, the *prmt1* gene in green anole is assigned to a separate scaffold of 222,408 bp (NW_003338834, Anolis carolinensis unplaced genomic scaffold, AnoCar2.0 chrUn0095, whole genome shotgun sequence) with *tbc1d17*, its human orthologue is a few genes downstream of the *cpt1c-tsks-ap2A-fuz-med25* cluster. The *bcl2L12* gene connected with *prmt1* in both human and alligator is located upstream of the *rcn3* and *med25* clusters in near proximity in green anole.

### Comparison of the PMRT8 sequences and gene arrangements close to *prmt8* in sauropsids

**Fig 4:**
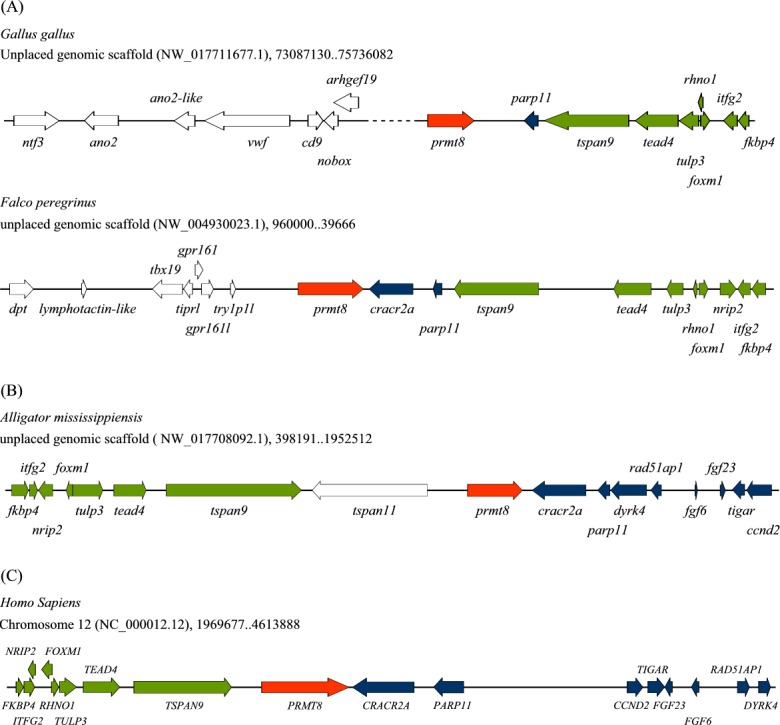
Syntenic gene arrangements near *prmt8* in amniotes. (A) Schematic diagram of the available gene arrangement around *prmt8* in chicken and peregrine falcon. (B) Schematic diagram of the available gene arrangement around *prmt8* in American alligator. (C) Gene arrangement around *prmt8* in human.

In comparison, we performed similar analyses for *prmt8*, the vertebrate paralogous gene of *prmt1*. From “gene” search in PubMed, thirty-one *prmt8* genes are annotated in birds and the *prmt8* gene is assigned to chromosome 1 in five different species including chicken, Japanese quail (*Coturnix japonica*), Great Tit (*Parus major*), turkey (*Meleagris gallopavo*) and zebra finch (*Taeniopygia guttata*). The avian PRMT8s also share very high sequence identities (~99%). Basically, the *prmt8* genes are in well assembled segments in avian genomes and the arrangements can be obtained in numerous species. We illustrated the syntenic gene arrangement around *prmt8* in chicken (Fig 4A). The arrangements of genes at the downstream side are conserved in avian species. However, the genes directly upstream of *prmt8* are completely different in chicken and falcons (Fig 4A) and other avian species.

When compared with gene arrangement in alligator, *cracr*2A-*parp11* downstream of *prmt8* in alligator fused with a syntenic block of eight genes from *tspan9* to *fkbp4* as in falcon. However, *tspan11*, the gene directly upstream of alligator *prmt8*, is missing in the avian species. The *cracr2A* gene directly downstream of *prmt8*, and the *nrip2* gene in the *tspan9* to *fkbp4* block, are missing in chicken but not in most other avian species (Fig 4B). A syntenic block of seven genes from *ccnd2* to *dyrk4* downstream of *prmt8* in alligator, though is not directly neighboring *prmt8* in chicken, is still present on the same chromosome around 2 Mbp upstream with *dyrk4* missing. This block is completely or partially reserved in synteny with *prmt8* in birds with different distances from *prmt8*.

We also examined the gene arrangements around *PRMT8* in human (Fig 4C). For the 19 genes from *ccnd2* to *fkbp4*, the only major differences between human and alligator are that *tspan11* in alligator is moved to further downstream of *PRMT8* in human, and the syntenic block of the seven genes from *ccnd2* to *dyrk4* in alligator flipped over in human.

### Expression of chicken *prmt8* at the RNA and protein levels

**Fig 5:**
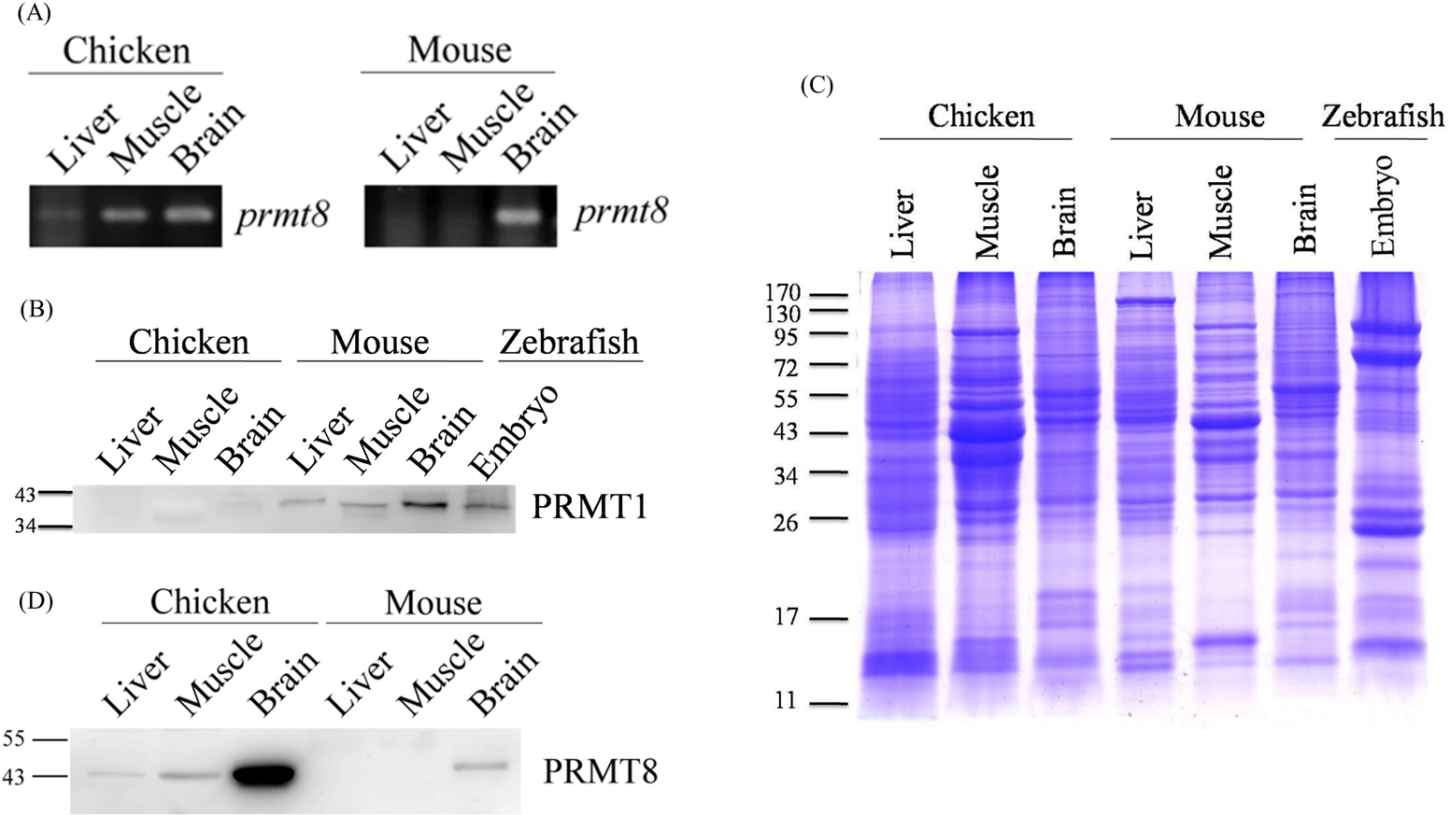
Expression analysis of *prmt1* and *prmt8* in chicken and mouse tissues. (A) Expression of *prmt8* transcripts in chicken and mouse tissues analyzed by RT-PCR. (B) Expression of PRMT1 protein in chicken and mouse tissues analyzed by western blots analysis. Proteins were prepared from chicken and mouse liver, muscle, and brain tissues. Zebrafish embryonic extract prepared from embryos 72 h post fertilization (hpf) was also included for comparison. Chicken and mouse tissue extracts as well as zebrafish 72 hpf embryonic extracts (25 μg) were examined using anti-PRMT1 (ab7027). (C) Chicken and mouse brain, liver, or muscle extracts as well as zebrafish 72 hpf embryonic extracts (25 μg) were analyzed by SDS-PAGE analysis and detected by coomassie stain. (D) Expression of PRMT8 protein in chicken and mouse tissues analyzed by western blots analysis. For PRMT8 analyses, chicken tissue extracts and mouse tissue extracts (25 μg) were examined using anti-PRMT8.

In mammals, PRMT1 is broadly expressed in different tissues and different PRMT1 isoforms function in cytosol and nucleus. In comparison, its paralogue PRMT8 is brain-specific and plasma membrane associated. As we confirmed the presence of chicken *prmt1* transcripts in Fig 1C, we also analyzed the expression of *prmt8* in chicken and mouse tissues by RT-PCR. As shown in Fig 5A, to our surprise, besides strong signals for *prmt8* transcripts in chicken brain, weak signals could be detected in chicken liver and muscle. However, mouse *prmt8* RNA was present in brain but not in liver or muscle as expected.

We then prepared brain, liver and muscle extracts from both chicken and mouse to examine the expression of PRMT1 and PRMT8 proteins. As shown in Fig 5B by western blot analyses, we detected weak signals in chicken brain and muscle but the signals detected in chicken muscle were lower than that of mouse PRMT1. Nevertheless, strong PRMT1 signals were detected in all mouse tissues. We also showed that the antibody (Abcam ab7027) could well recognize zebrafish PRMT1. Chicken PRMT1 shares 96% while zebrafish shares 91% sequence identity with human PRMT1. Thus the weak/no chicken PRMT1 signals are less likely to be due to the inability of the PRMT1 antibody to recognize the chicken PRMT1 protein.

Furthermore, another antibody (Millipore/Upstate 07–404) that could well recognize zebrafish PRMT1 as shown in our previous study [14], detected strong PRMT1 signals in all three mouse tissues but no PRMT1 signals in chicken brain and liver, even when there were three times more chicken protein (75 μg) compared with mouse protein (25 μg). Again very weak signals detected in chicken muscle were lower than that of mouse PRMT1 (S1 Fig).

For different tissues, the amount of GAPDH and beta-actin varies as detected by the antibody and thus were difficult to be used as loading controls. Nevertheless, coomassie blue staining showed the similar amount of protein loading of the chicken, mouse or zebrafish samples. The same tissue showed similar protein expression patterns whether they were from chicken or mouse (Fig 5C).

For PRMT8, the western blot results were consistent with the RT-PCR results. Interestingly, the PRMT8 level in chicken brain was much higher than that in mouse brain. Besides, weak PRMT8 protein signals were detected in chicken liver and muscle (Fig 5D). In mouse, the PRMT8 signals could be detected in brain but not in liver or muscle.

### Asymmetric dimethylarginine protein patterns in mouse and chicken tissues

**Fig 6:**
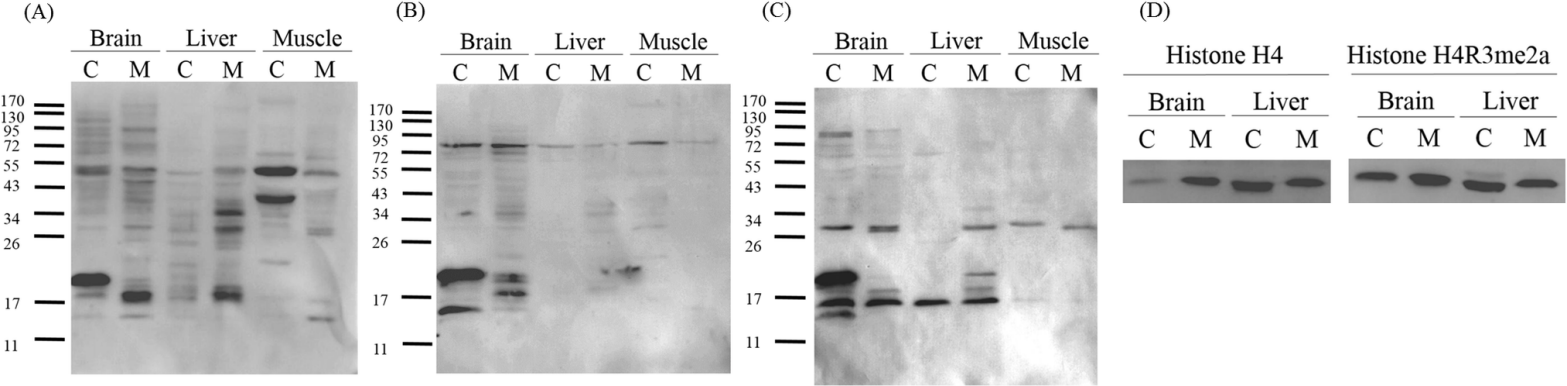
Methylarginine containing proteins in chicken and mouse tissue extracts. (A) Western blots analysis of chicken (C) and mouse (M) extracts using anti-ADMA antibodies. (B) Western blots analysis using anti-SDMA antibody. (C) Western blots analysis using anti-MMA antibodies. (D) Asymmetric dimethylation of histone H4 arginine 3 (H4R3) in chicken and mouse brain. Histones prepared from mouse and chicken brain extracts were analyzed by western with antibodies against histone H4 (left panel) and histone H4R3me2a (right panel).

Since PRMT1 is the major type I PRMT in other vertebrates but the expression level of chicken PRMT1 is low as shown in the previous section, we analyzed the levels of asymmetric dimethylarginine (ADMA) containing proteins formed by the catalysis of type I PRMTs in chicken. The levels of ADMA-containing proteins in chicken were close to that in mouse as detected by western blot analyses with ADMA-specific antibodies (Fig 6A). For brain and muscle extracts, the patterns of ADMA-containing proteins from the same tissue were similar whether they were from mouse or chicken. Nevertheless, two strong signals of the molecular mass of about 50 and 40 kDa were detected in chicken but not mouse muscle.

Birds retain type II and type III PRMTs catalyzing the formation of symmetric dimethylarginine (SDMA) and monomethylarginine (MMA). In comparison, we also analyzed the expression pattern of SDMA and MMA containing proteins with specific antibodies (Fig 6B and 6C). In general, SDMA and MMA signals in the same tissues were of similar levels and patterns whether they were from mouse or chicken.

Specifically, an intensive signal of the molecular mass around 20 kDa was detected in chicken but not mouse brain by all three methylarginine-specific antibodies (Fig 6A, 6B and 6C). A major polypeptide signal can be detected by protein staining at this molecular mass, indicating that it is highly abundant in chicken brain. We subjected this signal to mass spectrometric analyses for its identity. Multiple polypeptides with more than five peptides with the MASCOT score suggesting identity were identified including myelin basic protein that has been reported to contain SDMA [27].

We further detected the arginine methylation status of specific PRMT1 substrates in chicken. PRMT1 is an epigenetic modifier catalyzing the methylation of the arginine 3 (R3) residue of histone H4 to form H4R3Me2a [12]. We prepared histone proteins from both mouse and chicken brain. Specific H4R3 modification could be detected in chicken as well as in mouse brain using the H4R3Me2a-specific antibody (Fig 6D).

### Fractionation of the brain extract for subcellular distribution of type I PRMTs in chicken and mouse brain

**Fig 7:**
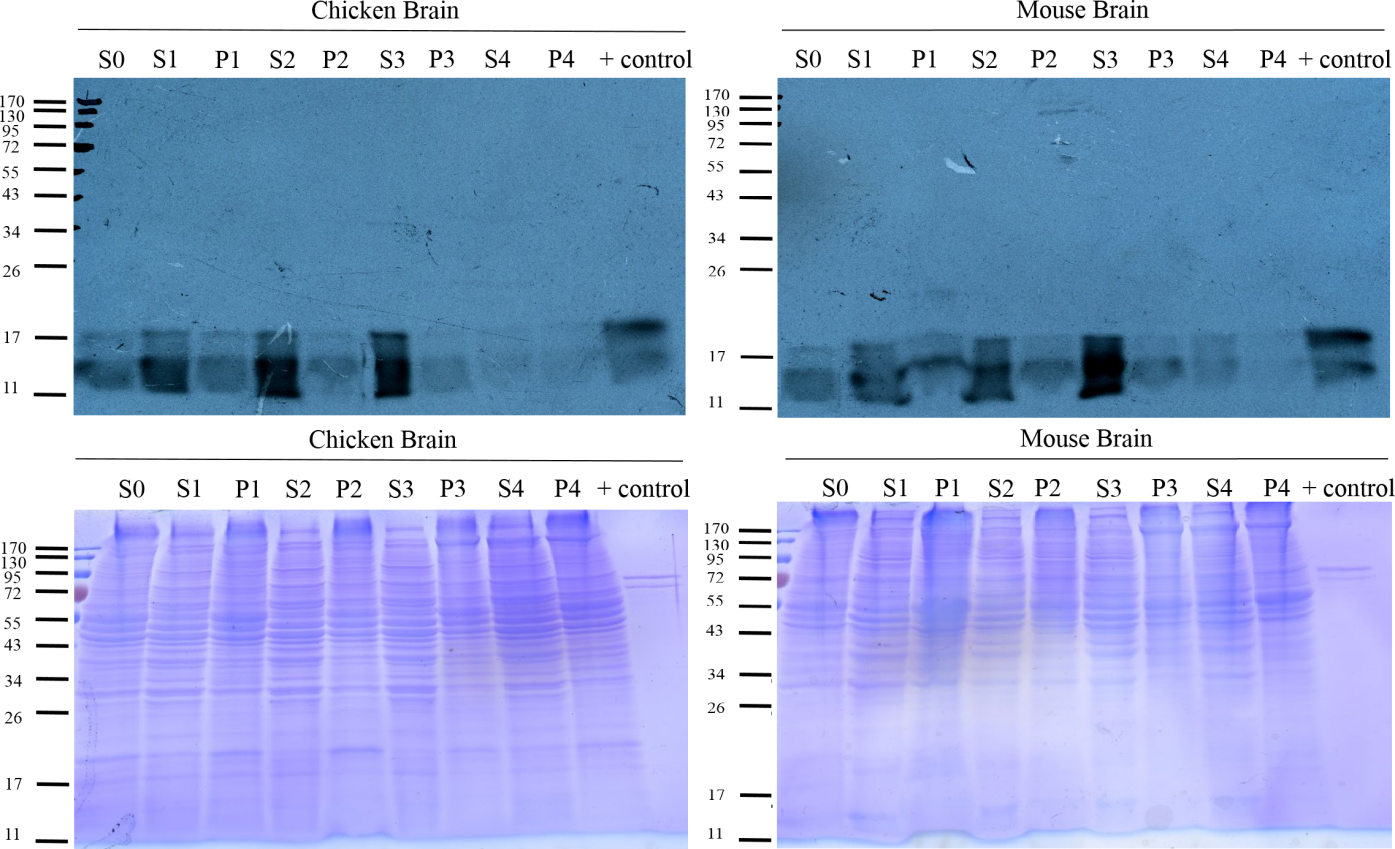
Type I protein arginine methyltransferase activity in chicken and mouse brain fractions. Analyses of the type I activity in the chicken (left panel) or mouse (right panel) brain fractions. *In vitro* protein methylation reactions were performed in the presence of various fractions (25 μg of protein), 1.5 μCi of the methyl group donor [^3^H] AdoMet, and with or without an arginine methyl-accepting substrate GAR to a final reaction volume of 15 μl. The reaction products were visualized by SDS-PAGE and fluorography. Recombinant GAR methylated by recombinant human PRMT1 was used as a positive control (+ control).

We would like to determine the PRMT activities in chicken. By *in vitro* methylation reactions, the methylation signals catalyzed by the tissue extracts were very low, with brain extract contained the highest PRMT activity. In case that there were inhibitors or certain factors in the total extracts that might interfere the assays, we further used a scheme similar to that had been used to fractionate porcine brain [24] to fractionate mouse and chicken brain extracts, and used the subcellular fractions as the enzyme source for methyltransferase assays (S2A Fig). The pellet from the first low-speed centrifugation (P1) is the nuclear fraction, and the pellet from the second high-speed centrifugation (P2) consists of large organelles such as mitochondria. The pellet from the ultracentrifugation (P3) contains membranes and super complexes such ribosomes, while the supernatant of the ultracentrifugation (S3) is cytosol. We used a recombinant glycine and arginine rich (GAR) protein with the GAR sequences from a typical PRMT substrate fibrillarin as the substrate. As shown in Fig 7, the supernatant fractions contained higher activity than the pellet fractions and the S3 fraction representing cytosol appeared to have the strongest type I PRMT activity in both mouse and chicken brain extract.

We also compared the distribution of PRMTs in the fractions (S2B Fig). PRMT1 signals were in all fractions of mouse brain extract but the strongest one was in the S4 fraction, the high salt stripped supernatant of the P3 fraction from ultracentrifugation. The peripheral association of mouse brain PRMT1 with membranes/ribosomes is similar to the PRMT1 distribution in the porcine brain [24]. Mouse and chicken PRMT8 showed similar expression pattern as mouse PRMT1.

We had examined the levels of *prmt3* transcripts in both chicken and mouse and observed that high expression level is high in brain and low in liver and muscle. We also detected the expression of PRMT3 in these brain fractions. PRMT3 in mouse and chicken was mainly detected in the S4 fraction. The result is consistent with the reports that PRMT3 exists only in the cytosol of mammalian cells [28] and methylate ribosomal protein S2 [29]. The distribution of PRMT5, the predominant type II enzyme catalyzing the formation of SDMA, was also detected. PRMT5 was present in all fractions and the strongest signals were in the P1 and P3 fractions. The S4 and P4 fractions both from the P3 fraction also showed high levels of PRMT5.

We finally detected some markers to verify the fractionation. Fragile X mental retardation protein (FMRP) and heterogeneous nuclear ribonucleoproteins A2/B1 (hnRNP A2/B1) showed the highest expression in S4. Besides, histone H3 was only detected in the P1 fraction, consistent with the nuclear distribution. These results confirmed the successful fractionation of the brain extracts.

## Discussion

In this study, we focused on the *prmt1* gene encoding the most conserved PRMT in birds. At first, we considered that this gene is missing in birds because we could not identify the gene in chicken genome, the earliest assembled and annotated avian genome assembly. In a previous study of conserved syntenic clusters of protein coding genes missing in birds, a block with genes just downstream of human *PRMT1* was recorded as a deletion group, and some genes just upstream of *PRMT1* were listed as missing genes in chicken in close proximity to missing syntenic blocks [21]. We thus speculated that a whole block of genes with *prmt1* was deleted in chicken. However, there are reports that genes previously considered missing in birds have been identified in avian species especially the recently sequenced and assembled genomes of saker falcon, perigrine falcon and ground tit [30]. Indeed, the *prmt1* gene was annotated in two falcons. We thus carefully examined the avian and chicken nucleotide databases and identified a few cDNA and EST targets encoding PRMT1. We assembled the sequences and predicted two chicken *prmt1* variants with high sequence identities with the human and alligator PRMT1. RT-PCR analyses confirmed the chicken *prmt1* expression. Besides, we identified two separate small contigs for separate *prmt1* exons, both with high GC contents. It is consistent with the study by Hron et al that a subset of avian genes with high GC content and long GC stretches are underrepresented in genomic databases and thus are hidden but not missing in birds [31]. Thus the *prmt1* gene is present in chicken but genomic fragments containing the gene were poorly recovered and assembled.

Further BLAST analyses against avian genomes in the databases showed that the *prmt1* gene is fragmented and only single and few exons are present in short scaffolds in most avian genomes. We also tried to assemble *prmt1* in other avian species with the limited sequences available. Extremely high sequence identities within avian PRMT1s and with human or alligator PRMTs indicate that PRMT1 is highly conserved in amnions.

The slowly evolved crocodilians are proposed to be close to the common ancestor of the archosaurs group containing crocodilians, birds and the extinct dinosaurs. We compared gene arrangements neighboring *prmt1* in birds (falcons), reptiles (American alligator and green anole) and mammals (human). Only the *rcn3* gene linked to *prmt1* in alligator is conserved in falcon. On the other hand, the gene arrangements near *prmt1* in alligator is very close to that in human. A syntenic block of eight genes upstream and another syntenic block containing five genes downstream of *PRMT1* are conserved in human and alligator. In human, the *RCN3* gene downstream of *PRMT1* in alligator moved upstream of the *NOSIP-PRMT1* block and flipped its direction. Interestingly, most of the orthologues of alligator and human *PRMT1* neighbors are present on chromosome 6 in green anole, but the *prmt1* gene is assigned to a separate long contig. Besides, the *bcl2l12* gene connected with *prmt1* in both human and alligator is located upstream of the *rcn3* and *med25* clusters in near proximity in green anole. Thus the *rcn3* and *bcl2l12* genes together with *prmt1* are the major breakpoints for chromosome translocations through evolution from the common ancestor to birds, mammals, and green anoles.

In the recent Gallus_gallus-5.0 genome assembly with 1911 protein coding genes added, some human PRMT1 neighbors previously consider missing [21] have been re-assigned to separate scaffolds of unknown chromosomes or have been predicted in other avian species but not in chicken. Genes including *RCN3* remained in the missing gene list [22]. Nevertheless, we showed that *rcn3* exists in falcons just behind *prmt1*, thus is present in birds. Though the missing gene list in chicken is shrinking, it is apparent that genes near human *PRMT1* in a large syntenic block on human chromosome 19 are underrepresented in chicken and many avian genomes currently. Human chromosome 19 has the highest gene density with 1,461 protein-coding genes and 321 pseudogenes [32]. Chicken microchromosomes have been considered to be gene rich but are poorly assembled even in the recent Gallus_gallus-5.0. They have higher GC content than macrochromosomes [22]. An attractive proposal is that chicken orthologues of human PRMT1 together with its neighbors on chromosome 19 are on microchromosomes that are still missing in the current assembly. However, more investigations are required for the proposal.

Besides *PRMT1*, *CARM1* (*PRMT4*) is also on human chromosome 19. *PRMT1* and *CARM1* locate on chromosome 19q13.3 and 19p13.2 respectively and both locations are reported to have syntenic blocks deleted in birds [21]. CARM1 is critical in mouse embryogenesis and mouse with a targeted disruption of CARM1 are small in size and die perinatally, thus is essential in mammals [33]. As reported by the recent Gallus_gallus-5.0, *carm1* can be predicted in eagle with correct synteny but does not BLAST align to chicken [22]. From gene search, *carm1* is predicted in 50 different avian species but no in Galliformes. Nevertheless, we could amplify chicken *carm1* cDNA by RT-PCR with the primer sequences conserved in other avian species. Thus *carm1* is not missing in chicken and its expression and function in birds require further analyses.

We also performed similar analyses for the *prmt1* paralogous gene *prmt8*. Unlike *prmt1* with no chromosomal assignment, *prmt8* is well assembled in alligator and some avian species. The gene is located on chicken chromosome 1 with neighbors annotated. Genes neighboring *prmt8* in alligator are in a similar arrangement in a syntenic block downstream of *prmt8* in the avian species we examined. However, the direct neighboring genes upstream of *prmt8* are completely different in different avian species and alligator (Fig 4). The gene arrangements around *prmt8* in human and alligator are highly conserved with one gene transposition and one syntenic block turn over.

The amniotes divided into the ancestral lineages of mammals and reptiles about 320 million years ago (MYA). Archosaurs containing crocodilians and birds is one of the two major clades in reptiles diverged about 280 MYA. Birds originated from a theropod lineage more than 150 MYA [18]. As alligator was proposed to be the closest to the common ancestor of archosaurs, through analyzing the human, crocodilian, chicken and a few avian genes on chromosomes, we suggested that *prmt1* with its syntenic clusters might be specifically rearranged in the evolution of the avian lineage since 280 MYA. Though the data is limited, it appears that both *prmt1* and *prmt8* changes their neighbors (at least one side) in different avian lineages in the recent 150 million years. In contrast, their neighbors and arrangements between crocodiles and human are highly conserved since 320 MYA.

We conducted RT-PCR and western blot analyses to confirm the expression of PRMT1and PRMT8 in chicken as well as to compare the PRMT expression in chicken and mouse. To our surprise, it appears that the expressed protein level of PRMT1 in chicken is lower than that in mouse, but the level of PRMT8 is higher than that in mouse. While PRMT1 is the major type I PRMT in almost all vertebrate species, in chicken we could barely detect PRMT1 protein expression in the tissues we examined. The antibodies we used could recognize zebrafish PRMT1. Compared with chicken PRMT1, zebrafish PRMT1 is less conserved with human PRMT1 that is the immunogen of the antibodies. It is thus unlikely that the weak/none chicken PRMT1 signals was due to poor antibody recognition of chicken PRMT1. Because we could detect the mRNA expression of chicken *prmt1*, why and how *prmt1* is regulated at the posttranscriptional or translational level requires further experiments.

Previous analyses of PRMT1 knockdown/knockout animals or cells all showed a significant decrease of ADMA-containing proteins as detected by western blot analyses using ADMA-specific antibodies [14, 34, 35]. With the limited expression of PRMT1, the level of ADMA-containing proteins detected in the chicken samples was of the same level as that in mice. Furthermore, arginine 3 of histone H4, a PRMT1 specific substrate, was methylated in chicken as detected by an H4R3me2a-specific antibody. These results indicated that the type I PRMT and PRMT1-like enzymatic activity function well in chicken. Furthermore, using a recombinant GAR protein as the substrate, we detected a similar level of type I PRMT activity in mouse and chicken brain. Nevertheless, *in vitro* methylation analyses revealed that fractions containing the highest type I PRMTs activity in mouse and chicken were not in accord with any PRMT expression patterns we examined. Though GAR with glycine and arginine rich sequences from human fibrillarin protein is a typical type I PRMT substrates, we could not exclude the possibility that type II and III PRMTs might still recognize the protein and catalyzed the reaction.

For other PRMT to complement low PRMT1 levels in chicken, it has to function in the same subcellular compartments as other vertebrate PRMT1. Fractionation of the brain extracts from either chicken or mouse showed highly concentrated PRMT3 in a specific fraction consistent with the previous reports of cytoplasmic distribution of PRMT3 [28] with the strong association ribosomes [29]. Thus PRMT3 is less likely to compensate for the function of other PRMTs in chicken for its restricted subcellular distributions.

PRMT8 is a minor brain-specific paralogue of PRMT1 in other vertebrates [15]. However, we detected high PRMT8 expression in chicken brain. Interestingly, PRMT8 protein in chicken brain is of a much higher level compared with that in mouse brain. Chicken PRMT8 was highly expressed in brain and distributed in similar sub-fractions as mouse PRMT1 or PRMT8. In addition, weak *prmt8* RNA, as well as protein signals, could be detected in chicken liver and muscle, indicating putative de-repression of *prmt8* expression in these chicken tissues. PRMT8 had been shown to methylate histone H4 *in vitro* [15, 36]. Though membrane localization of PRMT8 due to N-terminal myristoylation had been shown [15], there was evidence of nuclear localization of endogenous PRMT8 by immunohistochemical analyses of mouse brain tissues or stem cell derived neuronal cells [37, 38]. PRMT8 had been considered as a chromatin modifier in neuronal differentiation [39] and had been shown to be an epigenetic regulator for synaptic maturation by chromatin immunoprecipitation [40]. Thus it is possible that the H4R3me2 modification detected in chicken samples could be modified by the small amount of PRMT1 and/or also PRMT8. However, more experiments are required to examine the postulation that PRMT8 might at least partially complement low PRMT1 expression in chicken. PRMT8 knockout mice study showed that PRMT8 has the phospholipase activity to regulate Purkinje cell dendritic arborization and motor coordination [16]. We could not exclude the possibility that it is the phospholipase activity but not the PRMT activity that is critical for PRMT8 and upregulates PRMT8 expression in birds.

In summery, we determined the cDNA sequence of chicken *prmt1* and showed that its coding sequence is highly conserved with that of other amniotic PRMT1. However, the chromosomal localization of chicken *prmt1* remained undetermined and the PRMT1 protein expression level we detected was low. It is not known why chicken maintained the *prmt1* gene but expressed a low level of this critical PRMT. Instead, *prmt8* that is on chromosome 1 in chicken expressed at a high level. Chicken thus appears to be a unique natural system with *prmt1* knocked down and PRMT8 overexpression. Our studies provide the basic bioinformatic as well as biochemical information for further investigation in birds to elucidate the critical functions of protein arginine methylation conserved and not conserved in vertebrates.

## Supporting information

S1 Table Presence of the *prmt1* gene in avian genomes. (XLSX)

S1 File Assembled partial *prmt1* sequences from ground tit (*Pseudopodoces humilis*), canary (*Serinus canaria*) and crow (*Corvus brachyrhynchos*). (TXT)

S2 File Alignments of assembled avian PRMT1 nucleotide sequences. The predicted protein-coding sequences of assembled *prmt1* from chicken, ground tit, saker falcon, peregrine falcon, canary and crow are aligned with human and alligator PRMT1 by ClustalW.
(PDF)

S1 Fig Expression analysis of PRMT1 in chicken and mouse tissues. Expression of PRMT1 protein in chicken and mouse tissues analyzed by western blots analysis. Proteins were prepared from chicken and mouse liver, muscle and brain tissues. For PRMT1 analyses, chicken tissue extracts (75 μg) and mouse tissue extracts (25 μg) were examined using anti-PRMT1 (Millipore/Upstate 07–404).
(TIF)

S2 Fig Distribution of type I PRMT proteins and methylarginine accepting proteins in the chicken and mouse brain fractions. (A) Fractionation of the brain was conducted as described in [24]. Basically, the brain was disrupted by homogenization in lysis buffer and the brain homogenate was then fractionated by sequential centrifugations. The P3 fraction was resuspended in lysis buffer with 0.5 M KCl and subjected to ultracentrifuge again to obtain S4 and P4. The fractionation procedures are illustrated, with S indicating supernatant and P indicating pellet. (B) Subcellular fractions of chicken or mouse brain extracts (30 μg) were resolved by SDS-PAGE, transferred to nitrocellulose membrane, and then analyzed by western blot analyses as described in the Materials and Methods.
(TIF)
