# Supplementary material for: Identification, chromosomal arrangements and expression analyses of the evolutionarily conserved prmt1 gene in chicken in comparison with its vertebrate paralogue prmt8
Source: PLoS One. 2017 Sep 21;12(9):e0185042. doi: 10.1371/journal.pone.0185042 (PMC5608299; doi:10.1371/journal.pone.0185042)
Supplement: S2 File — The predicted protein-coding sequences of assembled prmt1 from chicken, ground tit, saker falcon, peregrine falcon, canary and crow are aligned with human and alligator PRMT1 by ClustalW. (PDF) [file pone.0185042.s003.pdf]

```
                             10        20        30        40        50        60        70        80        90       100
                    ....|....|....|....|....|....|....|....|....|....|....|....|....|....|....|....|....|....|....|....|
Human V1            ATGGCGGCAGCCGAGGCCGCGAACTGCATCATGGAGAATTTTGTAGCCACCTTGGCTAATGGGATGAGCCTCCAGCCGCCTCTTGAAGAAGTGTCCTGTG
American alligator  ATGGCGG------AGGCCGCCAACTGCATCATGGAGACGTTTGTCACCACGCTGGCTAACGGGATGAGCCTGCGGACTGCGCTAGAAGATGTTTCGTGCA
Chicken V1          ATGGCGG------AGGCGGCGAACTGCATCATGGAG------------------------------------------------------GTTTCGTGCC
Chicken V2          ATGGCGG------AGGCGGCGAACTGCATCATGGAGAGCTTTGTAGCCACCCTGGCTAACGGCATGAGCCTGCAGACGGCGCGGGATGAAGTTTCGTGCC
Ground tit          ATGGCGG------AGGCGGCCAATTGTATCATGGAG------------------------------------------------------GTCTCCTGCC
Saker falcon        ATGGGGCCCCCCGAC-----------------------GTTTGTAGCCACCCTGGCTAACGGGATGAGCCTCCAGACGCCGCTAGAAGATGTTTCGTGCC
Peregrine falcon    ATGAGCC---------------------------------------------------------------TCCAGACGCCGCTAGAAGATGTTTCGTGCC
Canary              ----------------------------------------------------------------------------------------------------
Crow                ----------------------------------------------------------------------------------------------------

                            110       120       130       140       150       160       170       180       190       200
                    ....|....|....|....|....|....|....|....|....|....|....|....|....|....|....|....|....|....|....|....|
Human V1            GCCAGGCGGAAAGCAGTGAGAAGCCCAACGCTGAGGACATGACATCCAAAGATTACTACTTTGACTCCTACGCACACTTTGGCATCCACGAGGAGATGCT
American alligator  CCCAGCCTGAGGGCAGTGGGAAGCCCCCGGCTGAGGAGATGACATCCAAGGACTACTACTTCGACTCCTATGCCCACTTTGGCATCCACGAGGAGATGCT
Chicken V1          CCCCCGCGGAGAGCAGCTCCAAGCCGTCGGCAGAGGAGATGACCTCCAAGGATTACTACTTCGACTCCTACGCGCACTTCGGCATCCATGAGGAGATGCT
Chicken V2          CCCCCGCGGAGAGCAGCTCCAAGCCGTCGGCAGAGGAGATGACCTCCAAGGATTACTACTTCGACTCCTACGCGCACTTCGGCATCCATGAGGAGATGCT
Ground tit          CCCCCGGGGAGGGCAGCGCCAAACCCTCGGCCGAGGACATGACCTCCAAGGATTATTACTTCGACTCCTACGCACACTTCGGGATCCACGAGGAGATGCT
Saker falcon        CCCCCGGGGAGAGCAGCACCAAGCCCTCGGCCGAGGAGATGACCTCCAAGGATTACTACTTTGACTCCTACGCCCACTTTGGCATCCATGAGGAGATGCT
Peregrine falcon    CCCCCGGGGAGAGCAGCACCAAGCCCTCGGCCGAGGAGATGACCTCCAAGGATTACTACTTTGACTCCTACGCCCACTTTGGCATCCATGAGGAGATGCT
Canary              ------------------CCAAGCCCTCGGCCGAGGACATGACCTCCAAGGATTACTACTTCGACTCCTACGCCCACTTCGGCATCCACGAGGAGATGCT
Crow                -------------------------------------------------------------------------------------------GGAGATGCT

                            210       220       230       240       250       260       270       280       290       300
                    ....|....|....|....|....|....|....|....|....|....|....|....|....|....|....|....|....|....|....|....|
Human V1            GAAGGACGAGGTGCGCACCCTCACTTACCGCAACTCCATGTTTCATAACCGGCACCTCTTCAAGGACAAGGTGGTGCTGGACGTCGGCTCGGGCACCGGC
American alligator  GAAGGATGAGGTTCGCACCCTGACCTACAGGAACTCCATGTTCCACAACCGGCACCTCTTCAAGGACAAAGTAGTCTTGGATGTGGGCAGTGGCACTGGC
Chicken V1          GAAGGATGAGGTGCGCACGCTCACCTACCGCAACTCCATGTTCCACAACCGCCATCTGTTCAAGGACAAAGTGGTGCTGGACGTGGGCAGCGGCACCGGC
Chicken V2          GAAGGATGAGGTGCGCACGCTCACCTACCGCAACTCCATGTTCCACAACCGCCATCTGTTCAAGGACAAAGTGGTGCTGGACGTGGGCAGCGGCACCGGC
Ground tit          GAAGGACGAGGTTCGCACTCTCACCTACCGCAACTCCATGTTCCACAACCGACACCTGTTCAAGGACAAGGTGGTGCTGGACGTGGGCAGCGGCACCGGC
Saker falcon        GAAGGATGAAGTGCGGACGCTGACCTACCGCAACTCCATGTTCCACAACCGGCACCTCTTCAAGGACAAAGTGGTGCTGGATGTCGGCAGCGGCACTGGC
Peregrine falcon    GAAGGATGAAGTGCGGACGCTGACCTACCGCAACTCCATGTTCCACAACCGGCACCTCTTCAAGGACAAAGTGGTGCTGGATGTCGGCAGCGGCACTGGC
Canary              GAAGGACGAGGTGAGGACGCTGACCTACCGCAACTCCATGTTCCACAACCGGCACCTGTTCAAGGACAAGGTGGTGCTGGACA-----------------
Crow                GAAGGACGAGGTGCGGACGCTGACCTACCGCAACTCCATGTTCCACAACCGGCACCTGTTCAAGGACAAGGTGGTGCTGGACGTGGGCAGCGGCACCGGC
```

```
                             310       320       330       340       350       360       370       380       390       400
                    ....|....|....|....|....|....|....|....|....|....|....|....|....|....|....|....|....|....|....|....|
Human V1            ATCCTCTGCATGTTTGCTGCCAAGGCCGGGGCCCGCAAGGTCATCGGGATCGAGTGTTCCAGTATCTCTGATTATGCGGTGAAGATCGTCAAAGCCAACA
American alligator  ATTCTCTGCATGTTTGCTGCCAAAGCAGGGGCTAAGCGTGTCATTGGGATCGAGTGCTCCAGCATCTCTGACTATGCCGTCAAGATTGTCAAAGCCAACA
Chicken V1          ATCCTATGCATGTTTGCTGCCAAGGCTGGGGCCCGGAGGGTCATCGGGATCGAGTGCTCCAGCATCTCCGATTACGCCGTCAAGATCGTCAAAGCCAACA
Chicken V2          ATCCTATGCATGTTTGCTGCCAAGGCTGGGGCCCGGAGGGTCATCGGGATCGAGTGCTCCAGCATCTCCGATTACGCCGTCAAGATCGTCAAAGCCAACA
Ground tit          ATCCTCTGCATGTTCGCTGCCAAGGCCGGCGCCAGGAGGGTCATCGGGATCGAGTGCTCGAGCATTTCCGACTACGCCGTCAAGATCGTCAAGGCCAACA
Saker falcon        ATTCTCTGTATGTTTGCTGCCAAAGCTGGTGCCCGCCGCGTCATCGGGATTGAGTGCTCCAGTATTTCCGATTATGCCGTCAAGATAGTCAAGGCCAATA
Peregrine falcon    ATTCTCTGTATGTTTGCTGCCAAAGCTGGTGCCCGCCGCGTCATCGGGATTGAGTGCTCCAGTATTTCCGATTATGCCGTCAAGATAGTCAAGGCCAATA
Canary              ----------------------------------------------------------------------------------------------------
Crow                ATCCTCTGCATGTTCGCTGCCAAGGCCGGGGCCCGCAGGGTCATCGGGATTGAGTGCTCGAGCATCTCCGACTACGCCGTCAAGATCGTCAAGGCCAACA

                             410       420       430       440       450       460       470       480       490       500
                    ....|....|....|....|....|....|....|....|....|....|....|....|....|....|....|....|....|....|....|....|
Human V1            AGTTAGACCACGTGGTGACCATCATCAAGGGGAAGGTGGAGGAGGTGGAGCTCCCAGTGGAGAAGGTGGACATCATCATCAGCGAGTGGATGGGCTACTG
American alligator  AGCTGGACCATGTGGTATCCATCATCAAGGGGAAGGTGGAAGAGGTAGAGCTGCCGGTGGAGAAGGTAGACATCATCATCAGTGAGTGGATGGGCTACTG
Chicken V1          AATTGGACCATGTGGTGTCCATCATTAAGGGGAAGGTGGAGGAGGTGGAGCTGCCCGTGGACAAAGTGGACATCATCATCAGTGAGTGGATGGGCTACTG
Chicken V2          AATTGGACCATGTGGTGTCCATCATTAAGGGGAAGGTGGAGGAGGTGGAGCTGCCCGTGGACAAAGTGGACATCATCATCAGTGAGTGGATGGGCTACTG
Ground tit          AACTGGACCACGTGGTTTCCATCATCAAGGGCAAGGTGGAGGAGGTGGAGCTGCCGGTGGAGAAGGTCGACATCATCATCAGCGAGTGGATGGGATATTG
Saker falcon        AGCTGGACCATGTGGTCTCTATTATCAAAGGGAAGGTGGAGGAGGTGGAACTGCCAGTGGAGAAGGTCGACATCATCATCAGCGAGTGGATGGGCTACTG
Peregrine falcon    AGCTGGACCATGTGGTCTCTATTATCAAAGGGAAGGTGGAGGAGGTGGAACTGCCAGTGGAGAAGGTCGACATCATCATCAGCGAGTGGATGGGCTACTG
Canary              -----------GTGGTTTCCATCATCAAGGGCAAGGTGGAGGAGGTGGAGCTGCCCGTGGACAAGGTTGACATCATCATCAGCGAGTGGATGGGCTATTG
Crow                AACTGGACCAAGTGGTTTCCATCATCAAGGGCAAGGTGGAGGAGGTGGAGCTGCCGGTGGAGAAGGTCGACATCATCATCAGCGAGTGGATGGGCTACTG

                             510       520       530       540       550       560       570       580       590       600
                    ....|....|....|....|....|....|....|....|....|....|....|....|....|....|....|....|....|....|....|....|
Human V1            CCTCTTCTACGAGTCCATGCTCAACACCGTGCTCTATGCCCGGGACAAGTGGCTGGCGCCCGATGGCCTCATCTTCCCAGACCGGGCCACGCTGTATGTG
American alligator  CCTCTTCTATGAGTCCATGCTCAACACTGTCATCTACGCCCGTGACAAGTGGCTGACCCCAGATGGACTCATCTTCCCAGACCGTGCCACGCTGTATGTT
Chicken V1          CCTGTTCTACGAGTCCATGCTCAACACCGTCATCTACGCGCGCGACAAGTGGCTGACCCCCGACGGCCTCATCTTCCCCGACCGCGCCACGCTGTATGTG
Chicken V2          CCTGTTCTACGAGTCCATGCTCAACACCGTCATCTACGCGCGCGACAAGTGGCTGACCCCCGACGGCCTCATCTTCCCCGACCGCGCCACGCTGTATGTG
Ground tit          TCTGTTCTACGAGTCCATGCTCAACACCGTCATCTACGCCCGCGACAAGTGGCTA-CCCCGGACGGGCTGATCTTCCCCGACCGCGCCACGCTCTACGTG
Saker falcon        CCTCTTCTACGAGTCCATGCTCAACACAGTCATCTACGCTCGTGACAAGTGGCTGACCCCTGACGGCCTCATCTTCCCTGACCGGGCAACGCTCTATGTG
Peregrine falcon    CCTCTTCTACGAGTCCATGCTCAACACAGTCATCTACGCTCGTGACAAGTGGCTGACCCCTGACGGCCTCATCTTCCCTGACCGGGCAACGCTCTATGTG
Canary              TCTGTTCTACGAGTCCATGCTCAACACCGTCATCTACGCCAGGGACAAGTGGCTGAGCCCGGCCGGGCTGATCTTCCCCGACCGCGCCACGCTCTACGTG
Crow                CCTGTTCTACGAGTCCATGCTGAACACAGTCATCTATGCCCGGGATAAATGGCTG---------------------------------------------
```

```
                             610       620       630       640       650       660       670       680       690       700
                    ....|....|....|....|....|....|....|....|....|....|....|....|....|....|....|....|....|....|....|....|
Human V1            ACGGCCATCGAGGACCGGCAGTACAAAGACTACAAGATCCACTGGTGGGAGAACGTGTATGGCTTCGACATGTCTTGCATCAAAGATGTGGCCATTAAGG
American alligator  ACGGCCATTGAAGACCGGCAGTATAAGGACTACAAGATCCACTGGTGGGAGAACGTGTATGGCTTTGACATGTCGTGCATCAAGGACGTGGCCATCAAGG
Chicken V1          ACGGCCATTGAGGACCGGCAGTATAAGGACTACAAAATCCACTGGTGGGAGAACGTGTACGGCTTCGACATGTCGTGCATTAAGGACGTGGCCATCAAGG
Chicken V2          ACGGCCATTGAGGACCGGCAGTATAAGGACTACAAAATCCACTGGTGGGAGAACGTGTACGGCTTCGACATGTCGTGCATTAAGGACGTGGCCATCAAGG
Ground tit          ACGGCCATCGAGGACCGGCAGTACAAGGACTACAAAATCCACTGGTGGGAGAACGTTTACGGCTTCGACATGTCCTGCATCAAGGACGTGGCCATCAAGG
Saker falcon        ACAGCCATAGAAGACCGGCAGTACAAGGACTACAAGATCCACTGGTGGGAGAACGTCTACGGCTTTGACATGTCCTGCATCAAGGACGTGGCCATCAAGG
Peregrine falcon    ACAGCCATAGAAGACCGGCAGTACAAGGACTACAAGATCCACTGGTGGGAGAACGTCTACGGCTTTGACATGTCCTGCATCAAGGACGTGGCCATCAAGG
Canary              ACGGCCATCGAGGACCGGCAGTACAAGGACTACAAGATCCACTGGTGGGAGAACGTTTATGGCTTTGACATGTCCTGCATCAAGGACGTGGCCATCAAAG
Crow                -------------------------------------------GGTGGGAGAACGTCTACGGCTTCGACATGTCCTGCATCAAGGACGTGGCCATCAAGG

                             710       720       730       740       750       760       770       780       790       800
                    ....|....|....|....|....|....|....|....|....|....|....|....|....|....|....|....|....|....|....|....|
Human V1            AGCCCCTAGTGGATGTCGTGGACCCCAAACAGCTGGTCACCAACGCCTGCCTCATAAAGGAGGTGGACATCTATACCGTCAAGGTGGAAGACCTGACCTT
American alligator  AACCACTCGTGGATGTTGTAGACCCCAAGCAGCTGGTCACCAATGCCTGCCTCATCAAGGAGGTGGACATCTACACGGTGAAGGTGGAGGACCTGACATT
Chicken V1          AGCCCCTGGTGGACGTGGTGGACCCCAAGCAGCTCGTTACCAACGCCTGCCTCATTAAGGAGGTGGACATCTACACGGTGAAGGTGGAGGAGCTGACGTT
Chicken V2          AGCCCCTGGTGGACGTGGTGGACCCCAAGCAGCTCGTTACCAACGCCTGCCTCATTAAGGAGGTGGACATCTACACGGTGAAGGTGGAGGAGCTGACGTT
Ground tit          AGCCGCTGGTGGACGTGGTGGACCCCAAGCAGTTGGTGACCAACGCCTGCCTCATCAAGGAAGTGGACATCTACACGGTCAAGGTGGAGGAGCTGACCTT
Saker falcon        AGCCCCTTGTCGATGTGGTGGACCCTAAGCAGCTTGTTACCAATGCCTGCCTCATTAAGGAGGTGGACATCTACACGGTGAAGGTGGAGGAGCTGACCTT
Peregrine falcon    AGCCCCTTGTCGATGTGGTGGACCCTAAGCAGCTTGTTACCAATGCCTGCCTCATTAAGGTGGGGGACATCTACACGGTGAAGGTGGAGGAGCTGACCTT
Canary              AGCCCCTGGTGGACGTGGTGGACCCCAAGCAGCTGGTGACCAACGCCTGCCTCATCAAGGAAGTGGACATCTACACGGTGAAGGTGGAGGAGCTGACCTT
Crow                AGCCCCTGGTGGACGTGGTGGACCCCAAGCAGCTGGTGACCAACGCCTGCCTCATCAAGGAAGTCGACATCTACACGGTGAAGGTGGAGGAGCTGACGTT

                             810       820       830       840       850       860       870       880       890       900
                    ....|....|....|....|....|....|....|....|....|....|....|....|....|....|....|....|....|....|....|....|
Human V1            CACCTCCCCGTTCTGCCTGCAAGTGAAGCGGAATGACTACGTGCACGCCCTGGTGGCCTACTTCAACATCGAGTTCACACGCTGCCACAAGAGGACCGGC
American alligator  CACGTCACCCTTCTGCCTCCAAGTGAAGCGCAATGACTACATCCATGCCCTAGTGGCCTACTTCAACATTGAGTTCACTCGCTGTCACAAGCGCACCGGC
Chicken V1          CACGGCGCCGTTCTGCCTGCAGGTGAAGCGCAATGATTACGTCCACGCGCTCGTCGCCTACTTCAACATCGAGTTCACGCGCTGCCATAAGCGCACCGGC
Chicken V2          CACGGCGCCGTTCTGCCTGCAGGTGAAGCGCAATGATTACGTCCACGCGCTCGTCGCCTACTTCAACATCGAGTTCACGCGCTGCCATAAGCGCACCGGC
Ground tit          CACGGCGCCCTTCTGCCTGCAGGTGAAGCGCAACGATTACGTGCACGCCCTGGTCGCCTACTTCAACATCGAGTTCACGCGCTGCCACAAGCGCACCGGC
Saker falcon        CACGGCACCCTTCTGTCTCCAAGTCAAGCGCAACGACTATGTGCACGCGCTAGTGGCGTACTTCAACATCGAGTTCACGCGCTGCCACGGCATCCCTGCG
Peregrine falcon    CACGGCACCCTTCTGTCTCCAAGTCAAGCGCAACGACTATGTGCACGCGCTAGTGGCGTACTTCAACATCGAGTTCACGCGCTGCCACAAGCGCACGGGC
Canary              CACGGCGCCCTTCTGCCTGCAGGTGAAGCGCAACGATTACGTGCACGCGCTGGTGGCCTACTTCAACATCGAGTTCACCCGCTGCCACAAGAGAACCGGC
Crow                CACGGCGCCCTTCTGCCTGCAGGTGAAGCGCAACGATTACGTGCACGCCCTGGTCGCCTACTTCAACATCGAGTTCACGCGCTGCCACAAGCGCACCGGC
```

```
                            910       920       930       940       950       960       970       980       990      1000
                    ....|....|....|....|....|....|....|....|....|....|....|....|....|....|....|....|....|....|....|....|
Human V1            TTCTCCACCAGCCCCGAGTCCCCGTACACGCACTGGAAGCAGACGGTGTTCTACATGGAGGACTACCTGACCGTGAAGACGGGCGAGGAGATCTTCGGCA
American alligator  TTCTCCACCAGCCCTGAGTCCCCCTACACTCACTGGAAGCAGACAGTGTTCTACATGGAGGACTATCTGACAGTGAAGACAGGCGAGGAGATCTTTGGCA
Chicken V1          TTCTCCACCAGCCCGGAGTCTCCGTACACGCACTGGAAGCAGACGGTGTTCTACATGGAGGACTACCTGACCGTGAAGACCGGCGAGGAGATCTTCGGCA
Chicken V2          TTCTCCACCAGCCCGGAGTCTCCGTACACGCACTGGAAGCAGACGGTGTTCTACATGGAGGACTACCTGACCGTGAAGACCGGCGAGGAGATCTTCGGCA
Ground tit          TTCTCCACCAGCCCCGAGTCGCCGTACACGCACTGGAAGCAGACGGTGTTCTACATGGAGGAATACCTGACGGTGAAGAGCGGGGAGGAGATTTTCGGCA
Saker falcon        GTGTCCCCAGGCCCTGAGTCCCCCTACACACACTGGAAGCAGACTGTGTTCTACATGGAGGACTACCTCACCGTCAAGACTGGGGAGGAGATCTTTGGCA
Peregrine falcon    TTCTCCACCAGCCCCGAGTCTCCCTACACACACTGGAAGCAGACTGTGTTCTACATGGAGGACTACCTCACCGTCAAGACTGGGGAGGAGATCTTTGGCA
Canary              TTCTCCACCAGCCCCGAGTCCCCGTACACGCACTGGAAGCAGACGGTGTTCTACATGGAGGAATACCTGACGGTGAAGAGCGGCGAGGAAATCTTCGGCA
Crow                TTCTCCACCAGCCCCGAGTCGCCGTACACGCACTGGAAGCAGACCGTGTTCTACATGGAGGAGTACCTGACGGTGAAGAGCGGCGAGGAGATCTTCGGCA

                           1010      1020      1030      1040      1050      1060      1070      1080      1090      1100
                    ....|....|....|....|....|....|....|....|....|....|....|....|....|....|....|....|....|....|....|....|
Human V1            CCATCGGCATGCGGCCCAACGCCAAGAACAACCGGGACCTGGACTTCACCATCGACCTGGACTTCAAGGGCCAGCTGTGCGAGCTGTCCTGCTCCACCGA
American alligator  CCATCAGCATGAAACCCAATGCCAAGAACAATCGTGACCTGGACTTCACCATCGACCTGGACTTCAAGGGCCAGCTCTGTGAGCTGTCATGTTCCACGGA
Chicken V1          CCATCACCATGAAGCCCAACGCCAAGAACAACCGTGACCTCGACTTCACCATCGACCTGGACTTCAAGGGGCAGCTGTGTGAGCTCTCCTGCTCCACCGA
Chicken V2          CCATCACCATGAAGCCCAACGCCAAGAACAACCGTGACCTCGACTTCACCATCGACCTGGACTTCAAGGGGCAGCTGTGTGAGCTCTCCTGCTCCACCGA
Ground tit          CCATCACCATGAAACCCAACGCCAAAAACAACCGCGACCTGGATTTCACCATCGACCTGGACTTCAAGGGGCAGCTCTGCGAACTCTCGTGCTCCACCGA
Saker falcon        CCATCACCATGAAGCCCAACGCCAAGAACAACCGTGACCTTGACTTCACCATCGACCTGGACTTCAAGGGGCAGCTCTGTGAGCTCTCCTGCTCCACTGA
Peregrine falcon    CCATCACCATGAAGCCCAACGCCAAGAACAACCGTGACCTTGACTTCACCATCGACCTGGACTTCAAGGGGCAGCTCTGCGAGCTCTCCTGCTCCACTGA
Canary              CCATCACCATGAAACCCAACGCCAAAAACAACCGCGACCTGGATTTCACCATCGACCTGGACTTCAAGGGGCAGCTCTGTGAACTCTCCTGCTCCACCGA
Crow                CCATCACCATGAAACCCAACGCCAAAAACAACCGCGACCTGGACTTCACCATCGACCTGGACTTCAAGGGGCAGCTCTGCGAACTCTCCTGCTCCACCGA

                           1110
                    ....|....|....|.
Human V1            CTACCGGATGCGCTGA
American alligator  CTATCGGATGCGCTAG
Chicken V1          TTACCGCATGCGTTAG
Chicken V2          TTACCGCATGCGTTAG
Ground tit          CTACCGCATGCGTTAG
Saker falcon        CTACCGCATGCGCTAG
Peregrine falcon    CTACCGCATGCGCTAG
Canary              CTACCGCATGCGCTAG
Crow                CTACCGCATGCGTTA-
```
