# Supplementary figures and images for: Identification, chromosomal arrangements and expression analyses of the evolutionarily conserved prmt1 gene in chicken in comparison with its vertebrate paralogue prmt8
Source: PLoS One. 2017 Sep 21;12(9):e0185042. doi: 10.1371/journal.pone.0185042 (PMC5608299; doi:10.1371/journal.pone.0185042)

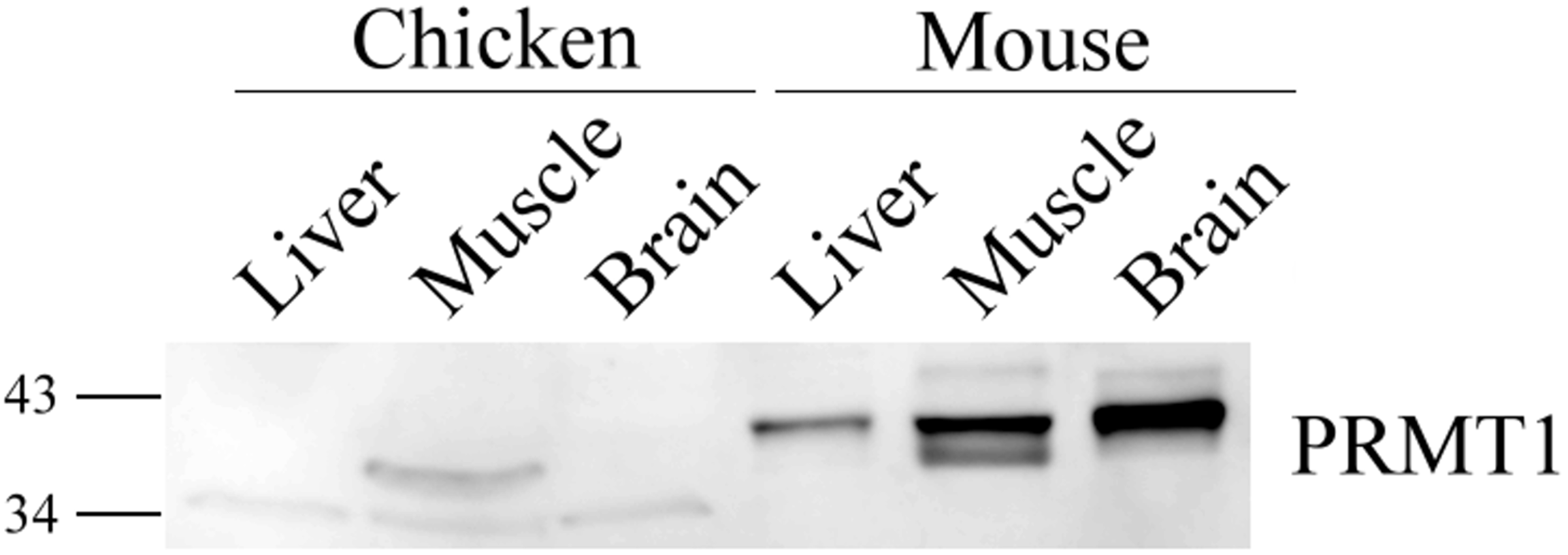

Supplement: S1 Fig — Expression of PRMT1 protein in chicken and mouse tissues analyzed by western blots analysis. Proteins were prepared from chicken and mouse liver, muscle and brain tissues. For PRMT1 analyses, chicken tissue extracts (75 μg) and mouse tissue extracts (25 μg) were examined using anti-PRMT1 (Millipore/Upstate 07–404). (TIF) [file pone.0185042.s004.tif]

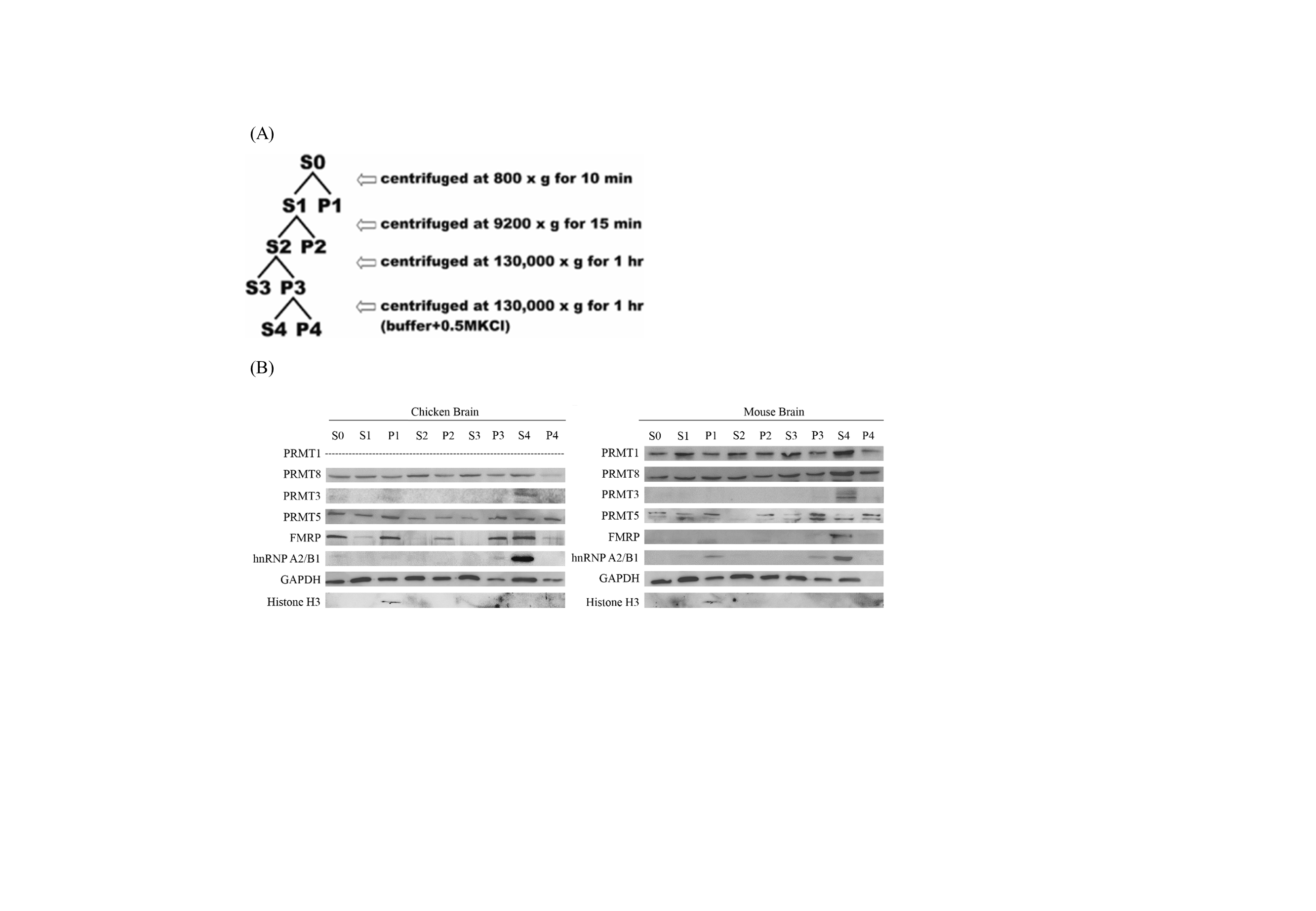

Supplement: S2 Fig — (A) Fractionation of the brain was conducted as described in [24]. Basically, the brain was disrupted by homogenization in lysis buffer and the brain homogenate was then fractionated by sequential centrifugations. The P3 fraction was resuspended in lysis buffer with 0.5 M KCl and subjected to ultracentrifuge again to obtain S4 and P4. The fractionation procedures are illustrated, with S indicating supernatant and P indicating pellet. (B) Subcellular fractions of chicken or mouse brain extracts (30 μg) were resolved by SDS-PAGE, transferred to nitrocellulose membrane, and then analyzed by western blot analyses as described in the Materials and Methods. (TIF) [file pone.0185042.s005.tif]
